# Pathways to Self-Compassion: The Effects of Self-Kind Behaviors and Self-Kind Thoughts on Self-Compassion and Well-Being

**DOI:** 10.1007/s42761-026-00379-4

**Published:** 2026-06-24

**Authors:** Ramona L. Martinez, Annie Regan, Sonja Lyubomirsky

**Affiliations:** 1 Department of Psychology, University of California, 900 University Ave.900 University Ave., Riverside, CA 92521 USA; 2 Department of Psychology, California State University, 1250 Bellflower Blvd., Long Beach, CA 90840-0901 USA

**Keywords:** Self-compassion, Well-being, Intervention, Ethnicity, First-generation college students

## Abstract

Self-compassion interventions often combine cognitive and behavioral exercises, making it challenging to identify intervention targets that drive self-compassion, and ultimately, well-being. Our 1-week experiment examined whether cognitive vs. behavioral self-kindness uniquely impacts self-compassion and well-being and whether pre-registered moderators (gender, ethnicity, first-generation college status) influence intervention effects. Undergraduates (*N* = 739) were randomized into one of three conditions: cognitive self-kindness (thinking about oneself kindly), behavioral self-kindness (performing kind acts for oneself), or active control (listing daily activities). Participants completed their assigned activities three times over 1 week. Participants in the behavioral self-kindness group reported greater posttest self-compassion compared to the cognitive self-kindness and control groups. Moderator analyses indicated that Latinx and first-generation college students uniquely benefited from exercising self-kindness—especially cognitive self-kindness. Being kind toward oneself may be a more effective short-term strategy to increase self-compassion compared to thinking kind thoughts about oneself, which might require more time and practice. Further research is needed to test cognitive self-compassion as an especially useful tool to bolster Latinx and first-generation college student well-being.

**Supplementary Information:**

The online version contains supplementary material available at https://doi.org/10.1007/s42761-026-00379-4.

Self-compassion—treating oneself kindly in the face of failure or suffering—has become a focus of psychological research in recent decades (Neff, 2003, 2023). According to a commonly-adopted definition (Neff, 2003), self-compassion includes three components: (a) self-kindness (treating oneself as a good friend would), (b) common humanity (recognizing failure and suffering as a part of the human experience), and (c) mindfulness (having nonjudgmental awareness of one’s suffering or failure instead of over-identifying with it). Converging research links self-compassion to greater psychological well-being, broadly defined, as well lower psychopathology (Breines & Chen, 2012; MacBeth & Gumley, 2012; Neff & Germer, 2013; Neff et al., 2007; Shapira & Mongrain, 2010), highlighting its significance for mental health and flourishing.

While meta-analytic evidence points to the positive relationship between self-compassion and psychological well-being generally (e.g., Ferrari et al., 2019; MacBeth & Gumley, 2012), more research is needed to clarify how demographic and cultural differences bear on the relationship between self-compassion and well-being. Some research indicates, for example, that self-compassion might be less closely tied to well-being among East Asians (e.g., Wang & Lou, 2022) but may be especially conducive to the well-being of Hispanics (e.g., Piña-Watson et al., 2025).

Moreover, while growing research suggests that college students with higher self-compassion generally report greater psychological well-being relative to their less self-compassionate peers (Kroshus et al., 2021; Neely et al., 2009), less is known about the potential benefits of self-compassion among specific student populations—especially those that may stand most to benefit from exercising self-kindness. For example, first-generation college students, whose parents did not attend or complete college, are especially likely to report relatively chronic academic, social, and financial stressors (Kroshus et al., 2021). Limited available research offers mixed findings as to whether self-compassion protects or bolsters the well-being of first-generation college students compared to continuing-generation students (e.g., Kroshus et al., 2021; Ramos Salazar & Meador, 2025), necessitating additional efforts to understand the dynamics of self-compassion among specific student demographics.

## Strategies for Developing the Components of Self-Compassion

Intervention research suggests that self-compassion can be cultivated through a range of approaches, from more intensive interventions including compassion-focused therapy (CFT) in clinical settings (Gilbert, 2010, 2014) and an 8-week, guided Mindfulness Based Self-Compassion (MSC) training program (Neff & Germer, 2013), to 1–2 week programs (Dundas et al., 2017; Shapira & Mongrain, 2010), and 3-minute microinterventions (Elefant et al., 2017). These self-compassion interventions are multimodal and include a range of training strategies to develop the components of self-compassion. For example, as part of these interventions, someone might develop self-kindness by practicing talking to oneself from the perspective of a kind friend (either as a thought exercise or by writing a self-addressed letter; Neff & Germer, 2013). Furthermore, a person may develop mindfulness through meditation, breathing exercises, and labeling emotions (Gilbert, 2014; Neff & Germer, 2013). Finally, a person might develop a sense of common humanity through perspective-taking exercises (Gilbert, 2014) or practicing self-compassion in community and discussion with others (Neff & Germer, 2013). Notably, while some self-compassion exercises largely focus on a single component of self-compassion, other exercises tap into multiple or all three self-compassion components, either all at once or in sequence. The “self-compassion break,” for example, is an exercise that invokes self-kindness, mindfulness, and common humanity through a series of reflective prompts (Germer & Neff, 2019). In the interest of maximizing well-being outcomes, a multimodal intervention approach that targets all self-compassion components seems well-justified, but from a basic scientific perspective, additional work is needed to clarify how developing these individual components drive self-compassion, and ultimately, well-being.

## Practicing the Component of Self-Kindness: Cognitive vs. Behavioral Strategies

Overall, self-compassion strategies are largely cognitive (reflective) in nature—for example, meditation, perspective-taking, and visual imagery. The self-kindness component of self-compassion especially relates to cultivating a way of thinking towards oneself that is caring, understanding, and warm, especially when confronted with personal shortcomings and challenging situations (Gilbert, 2014; Neff & Germer, 2013; Smith et al., 2018).

Although theoretical and empirical work on self-compassion in recent decades focuses mainly on exercising self-kindness as a way of thinking, self-kindness in times of distress might also include more behavioral acts of self-care. Research indicates, for example, that undergraduates tend to “treat themselves” by watching shows/movies, eating favorite foods or snacks, shopping, going to an event, or carrying out hobbies (sports, baking, playing an instrument, etc.) as a reward for doing well or as a comfort when things go wrong (Schiavone et al., 2023). Relatedly, other research encourages professionals in demanding fields, such as social work and therapy/counseling, to prioritize self-care behaviors (behaviors that promote subjective and physical well-being) to protect against chronic stress and burnout (Lee & Miller, 2013; Posluns & Gall, 2020). Such self-care behaviors include spending time on hobbies, rest, eating well, and exercise (Lee & Miller, 2013).

To date, little to no research examines whether relatively more behavioral acts of self-kindness also promote self-compassion. In addition, more studies are needed to further understand the potential well-being benefits of self-kind acts. Although some research suggests behavioral acts of self-kindness are conducive to well-being (e.g., self-care in professional settings), other experimental evidence suggests that only other-directed (prosocial) kind acts (e.g., cooking for someone else) promote psychological flourishing, while self-kind acts (e.g., going to the spa) do not (Nelson et al., 2016). Additional work is needed to clarify if and how behavioral self-kindness impacts well-being, as compared to cognitive self-kindness.

## Self-Compassion and Cultural Differences

Research is also needed to clarify how individual and cultural differences bear on the relationship between self-compassion and well-being. Converging evidence points to the broad efficacy of self-compassion interventions (e.g., Ferrari et al., 2019; Kirby et al., 2017), but little research examines relevant individual differences, such as race-ethnicity, that may moderate the relationship between self-compassion and target outcomes.

Limited but informative research offers insight into the relationship between self-compassion and well-being with respect to ethnic and cultural differences. Psychometric studies, for example, have replicated the factor structure of self-compassion across ethnicities and cultures among samples from North America, South America, Europe, East Asia, and Oceania (Cababie & Etchezahar, 2023; Neff et al., 2019; Zhang et al., 2019). Notably, there are relatively fewer psychometric validation studies among Latinx cultures and countries compared to other cultural/regional groups, with validated self-compassion scales available for Argentina and Brazil at present (Cababie & Etchezahar, 2023; Neff et al., 2019).

Other research points to functional differences in self-compassion across race-ethnicity. For example, in a study comparing the relationships between self-compassion and self-criticism among Asian American, European American, and Latinx participants in the northeast U.S., self-compassion and self-criticism were positively correlated among Asian American participants but negatively correlated among European American and Latinx participants (Boyraz et al., 2021). The authors argued that these differences reflect the capacity of members of Asian cultures to hold dialectical beliefs. These speculations are supported by other survey research that suggest that, although Chinese and U.S. undergraduates do not vary in overall self-compassion (as measured by composite scores), Chinese undergraduates report significantly higher subscale scores in both positive aspects of self-compassion (self-kindness and common humanity) and negative aspects of self-compassion (isolation and over-identification with failure; Birkett, 2014) relative to U.S. undergraduates. That is, across both these studies (Birkett, 2014; Boyraz et al., 2021), researchers observed that people of Asian heritage tend to hold both positive and negative aspects of self-regard in tandem.

In addition, meta-analytic evidence suggests there is a weaker relationship between self-compassion and life satisfaction among collectivist (e.g., East Asian) cultures compared to individualist cultures (Wang & Lou, 2022). Plausibly, the practice of thinking of oneself with a kinder lens may also be inconsistent the well-replicated finding that East Asians, relative to Westerners, are less likely to view and present themselves in a positively biased way (i.e., to self-enhance; Heine & Hamamura, 2007). That is, among East Asian cultures, where there is relatively less emphasis on self-concern and personal wellness, self-compassion might be less closely tied to well-being.

Relatively less is known about the functional benefits of self-compassion among Latinx populations, but some burgeoning research offers foundational knowledge in this area. For example, one survey study with over 800 Mexican–American college students found that self-compassion separately buffered the relationship between acculturation stress and guilt, anxiety, and suicide risk (Piña-Watson et al., 2025). Another survey study of over 2,000 parents in the U.S. carried out during the COVID-19 pandemic found that Hispanic parents reported relatively higher self-compassion than White parents, and that higher self-compassion conferred protective benefits on parent and child mental health among Hispanics (Kroshus et al., 2023). More research is needed to understand self-compassion dynamics and potential benefits among Latinx/Hispanic populations.

Some work also offers insights into the efficacy of self-compassion interventions for U.S.-based and global Latinx populations. Of note, a small pilot study (*N* = 20) of a Mindfulness-Based Stress Reduction for Teens program increased self-compassion and reduced depression among Latinx middle school students (Edwards et al., 2014). Relatedly, a meta-analysis on culturally-adapted mindfulness interventions suggests that such interventions significantly reduce distress among Hispanic participants in the U.S., Spain, and South America; although, only 10% of included studies specifically targeted self-compassion (Castellanos et al., 2020).

## Self-Compassion and Individual Differences in Undergraduate Well-Being

Emerging research suggests that self-compassion may be especially beneficial in protecting or boosting the well-being of college student populations, especially during difficult circumstances (e.g., during the COVID-19 pandemic; Ramos Salazar & Meador, 2025) and among historically marginalized groups in academia (e.g., female and racial-ethnic minorities, Johnson & Plisco, 2025; first-generation college students, Ramos Salazar & Meador, 2025). The transition to college itself includes many stressors, including academic challenges (e.g., lower than expected grades), social challenges (e.g., difficulties with romantic partners, roommates, or family members), and practical challenges (e.g., financial difficulties; Kroshus et al., 2021).

Notably, first-generation college students are especially likely to report greater chronic academic, social, and financial stressors relative to continuing-generation students (Kroshus et al., 2021). Moreover, longitudinal research suggests that, while first-generation and continuing generation students report comparable well-being at the start of college, first-generation students report significantly lower well-being over the course of the first year (Bowman, 2010).

Generally, survey studies find that students with higher self-compassion report stronger mental health and psychological adjustment (Kroshus et al., 2021; Neely et al., 2009), but little research offers insight into the relative benefits of self-compassion for first-generation college students compared to continuing-generation students. One cross-sectional study indicated that first-generation students reported higher self-compassion relative to continuing-generation students during the COVID-19 pandemic, and self-compassion related to greater active learning (e.g., higher engagement through class participation, use of discussion boards, etc.; Ramos Salazar & Meador, 2025). By contrast, a 1-year longitudinal study among first-year college students suggested that self-compassion did not buffer the effect of chronic stressors on well-being among first-generation students (Kroshus et al., 2021). Additional research, particularly longitudinal and experimental work, is needed to examine whether self-compassion confers benefits to college students generally and first-generation college students particularly.

## The Present Study

The present study investigated whether cognitive and behavioral acts of self-kindness differentially relate to self-compassion and well-being, and, on an exploratory basis, examined whether the effects of cognitive and behavioral self-kindness on self-compassion and well-being are moderated by individual and cultural differences. To these ends, we conducted a 1-week self-compassion intervention. Participants were randomized to one of three conditions and completed one of the following exercises three times over the course of 1 week (see Supplementary Materials for instructions):

1. Cognitive self-kindness: Complete a written self-compassion exercise that involves reflecting on a personal struggle and writing to oneself about the struggle from the perspective of a close friend (adapted from Neff & Germer, 2013).
2. Behavioral self-kindness: Identify and carry out behavioral acts of kindness for at least 15 minutes on a given day. Example behaviors provided included having a favorite meal, treating oneself to a massage, or spending time on a favorite hobby (adapted from Fritz et al., 2021; Nelson et al., 2016).
3. Active control: Keep track of daily activities carried out as part of one’s normal daily routine and create an outline of these daily activities (e.g., ate breakfast, went to work, went to a meeting, worked out at the gym, etc.; adapted from Fritz et al., 2021; Nelson et al., 2016)

Our pre-post outcomes included trait self-compassion; depression, anxiety, and stress; brooding rumination; life satisfaction; self-efficacy; self-worth; and trait self-esteem. Our daily outcomes included positive and negative affect and state self-compassion. Finally, our moderator variables (measured at pretest) included the following demographics: age, gender, ethnicity, international student status, and first-generation college student status.

The following pre-registered questions and hypotheses were addressed: (https://doi.org/10.17605/OSF.IO/VUDE6):

### Research Question 1: Does a Short, Self-Directed Self-Kindness Writing Exercise Improve Well-Being?

Hypothesis 1: Participants in the cognitive self-kindness condition will report increases in well-being (i.e., life satisfaction, positive affect) and decreases in brooding rumination and symptoms of depression and anxiety at posttest relative to those in the control condition.

### Research Question 2: Does the Type of Self-Kindness Exercise (Cognitive v. Behavioral) Impact Well-Being Outcomes?

Hypothesis 2: Participants assigned to perform cognitive self-kindness will report greater increases in self-compassion and greater decreases in brooding rumination and symptoms of depression and anxiety relative to those assigned to perform behavioral self-kindness.

Hypothesis 3: Participants assigned to perform cognitive self-kindness will report greater self-efficacy, self-worth, and self-esteem relative to those in each of the other conditions.

Hypothesis 4: Participants in both self-kindness conditions (collapsed) will report greater positive affect and life satisfaction and lower negative affect across the intervention period relative to those in the control condition.

### Exploratory Aim 1: Test Individual Difference Moderators of Intervention Effects

We tested our pre-registered moderators (gender, ethnicity, first-generation student status, and international student status) on an exploratory basis.

## Method

### Participants

Participants were undergraduate students recruited from a student subject pool at a large public university in Southern California (*N* = 739, *M*_age_ = 19.53, *SD* = 2.15, Female = 63%; Table 1). A plurality of participants identified as Asian (45.87%), and the remaining participants identified as Hispanic or Latinx (29.91%), more than one ethnicity (9.74%), White (6.22%), Black (3.65%), or other (3.79%). Most participants were in their first (43.3%) or second (30.04%) year of college, with smaller proportions of college juniors (15.83%) and seniors (10.42%). Nearly half (49.17%) of participants reported being a first-generation college student, and a small proportion reported being an international student (4.98%).

**Table 1:** Pretest sample characteristics

	Full Sample (*N* = 739)	Control (*N* = 245)	Behavioral Self-Kindness (*N* = 249)	Cognitive Self-Kindness (*N* = 245)
Age M(SD)	19.53 (2.15)	19.4 (1.55)	19.39 (2.04)	19.8 (2.71)
Ethnicity				
Asian	45.87%	45.71%	47.79%	44.08%
Hispanic/Latinx	29.91%	31.02%	27.71%	31.02%
More than one	9.74%	8.57%	10.04%	10.61%
White	6.22%	6.12%	5.62%	6.94%
Other	3.79%	4.08%	4.02%	3.27%
Black	3.65%	3.67%	4.02%	3.27%
Native American	0.14%	0.41%	–	–
No response	0.68%	0.41%	0.80%	0.82%
Class Year				
Freshman	43.30%	44.49%	46.59%	38.78%
Sophomore	30.04%	31.02%	30.12%	28.98%
Junior	15.83%	14.69%	15.66%	17.14%
Senior	10.42%	9.80%	7.23%	14.29%
No response	0.41%	–	0.40%	0.82%
Gender				
Female	62.79%	61.22%	62.65%	64.49%
Male	33.96%	35.92%	34.54%	31.43%
Non-binary/non-conforming	2.44%	2.45%	1.61%	3.27%
Prefer not to answer	0.14%	–	0.40%	–
Transgender	0.14%	–	0.40%	–
No response	0.54%	0.41%	0.40%	0.82%
First-Generation Student	49.17%	50.46%	47.64%	49.43%
International Student	4.98%	5.65%	4.45%	4.84%

*M* = Mean; *SD* = Standard Deviation. Conditions did not significantly differ for any demographic variables (*p* >.05)

A power analysis conducted using G*Power suggested that a target sample size of at least 244 participants is required at posttest to detect small to medium between-condition differences in pretest to posttest analyses at 80% power. We overrecruited participants at pretest in anticipation of data exclusions and attrition across the intervention period. The overall attrition rate from pretest to posttest was 35.20%. Attrition did not differ by condition (χ^2^[2] = 2.92, *p* =.23). Results from a series of logistic regression models suggest that missing data were not predicted by demographic characteristics, including gender, age, ethnicity, or first-generation status (all *p*-values >.05). To equalize demand characteristics across conditions, all participants (including those in the active control condition) were told that their assigned activities could improve their well-being.

We preregistered two exclusion criteria: incomplete cases and fast responding. We removed eight cases across all timepoints where participants completed surveys faster than one item per second (Wood et al., 2003). Twelve cases were removed due to incomplete data (i.e., completed 50% or less of the questions in a given survey). Exclusion criteria were applied at each timepoint rather than removing participants from all analyses. After applying preregistered data exclusion criteria, a total of 475 participants completed all four timepoints.

### Measures

The online study had four major timepoints: T_1_ (Day 1/pretest): pretest measurements, demographics, traits, intervention exercise, daily outcomes; T_2_ (Day 3): intervention exercise, daily outcomes; T_3_ (Day 5): intervention exercise, daily outcomes; and T_4_ (Day 7/posttest): daily outcomes, posttest measurements.

#### Pre-Post Outcomes

##### Self-Compassion

Trait self-compassion was measured using the 26-item Self-Compassion Scale (Neff, 2003). Participants were asked to respond on a 5-point Likert type scale from 1 (*almost never*) to *5* (*almost always*). Sample items include*:* “I’m tolerant of my own flaws and inadequacies,” and “When I feel inadequate in some way, I try to remind myself that feelings of inadequacy are shared by most people.” The SCS was highly reliable across the duration of the study (T_1_ ω = 0.93, T_4_ ω = 0.94).

##### Depression, Anxiety, and Stress

Symptoms of depression, anxiety, and stress were measured using the 21-item Depression Anxiety and Stress Scales (DASS-21; Lovibond & Lovibond, 1995). The DASS-21 includes items such as “I couldn’t seem to experience any positive feeling at all” (depression subscale), “I felt I was close to panic” (anxiety subscale), and “I found it hard to wind down” (stress subscale). At pretest (T1), participants were asked to report how often each item applied to them over the previous week, on a scale from 0 (*did not apply to me at all*) to 3 (*applied to me very much, or most of the time*). At the posttest assessment (T4), participants reported how often each item applied to them since they began participating in the study. All subscales demonstrated good reliability across the duration of the study (depression: T1 ω = 0.93, T4 ω = 0. 93; anxiety: T1 ω = 0.84, T4 ω = 0. 87; stress: T1 ω = 0.87, T4 ω = 0. 89).

##### Brooding Rumination

Participants completed the 5-item brooding rumination subscale of the Rumination Response Scale (RRS; Treynor et al., 2003). Participants were asked how often they engaged in each of the five brooding rumination items on a scale from 1 (*almost never*) to 4 (*almost always*) over the previous week. Sample items include “Think ‘Why can’t I handle things better?’” and “Think about a recent situation, wishing it had gone better.” The RRS demonstrated good reliability across the duration of the study (T_1_ ω = 0.83, T_4_ ω = 0.83).

##### Life Satisfaction

Life satisfaction was measured using the 3-item life satisfaction subscale of the Comprehensive Inventory of Thriving (CIT; Su et al., 2014). The full CIT includes 54 items and 18 subscales that represent distinct dimensions of psychological and social functioning and was developed as a broad and holistic measure of well-being. All items in the CIT are rated on a scale from 1 (*strongly disagree*) to 5 (*strongly agree*). The life satisfaction subscale includes face-valid items such as “I am satisfied with my life.” This subscale demonstrated good reliability across the duration of the study (T_1_ ω = 0.86; T_4_ ω = 0.85).

##### Self-Efficacy

Self-efficacy was measured through the 3-item self-efficacy subscale of the CIT (Su et al., 2014), which includes items such as “I am confident that I can deal with unexpected events,” rated on the same 1 to 5 scale as the other CIT subscales. The self-efficacy subscale of the CIT demonstrated good reliability across the duration of the study (T_1_ ω = 0.79; T_4_ ω = 0.82).

##### Self-Worth

Self-worth was measured through the 3-item subscale of the CIT (Su et al., 2014), which includes items such as “What I do in life is valuable and worthwhile” (again, rated on a 1 to 5 scale). The self-worth subscale of the CIT demonstrated good reliability across the duration of the study (T_1_ ω = 0.87; T_4_ ω = 0.87).

##### Self-Esteem

Self-esteem was measured as an exploratory outcome using a modified 6-item version of the 10-item Rosenberg Self-Esteem Scale (RSE; Rosenberg, 1965). Participants responded to items such as “I am able to do things as well as most other people” and “On the whole, I’m satisfied with myself” on a scale from 1 (*strongly disagree*) to 5 (*strongly agree*) at T_1_ and T_4_. The RSE was highly reliable across the duration of the study (T_1_ ω = 0.90; T_4_ ω = 0.92).

#### Pretest Measures

##### Demographic Variables

Participants responded to a series of demographic questions, including age, gender identity, ethnicity, year in college, and four yes–no questions about their student status (international, first-generation, commuter, fraternity/sorority member).

##### Trait Rumination

Trait rumination was measured using the Rumination-Reflection Questionnaire (RRQ; (Trapnell & Campbell, 1999). The RRQ is a 23-item individual difference measure that distinguishes between reflective and ruminative self-awareness. Participants rated their agreement with each item on a scale from 1 (*strongly disagree*) to 5 (*strongly agree*). Sample items include “My attention is often focused on aspects of myself I wish I'd stop thinking about” (ruminative self-awareness), and “I love exploring my ‘inner’ self” (reflective self-awareness). Both subscales demonstrated good reliability (ruminative self-awareness ω = 0.92; reflective self-awareness ω = 0.92).

#### Daily Outcomes

The following measures were administered at all four timepoints: T_1_ (Day 1/pretest), T_2_ (Day 3), T_3_ (Day 5), and T_4_ (Day 7/posttest).

##### Positive and Negative Affect

Positive and negative affect were measured using a modified version of the Affect-Adjective Scale (AAS; (Diener & Emmons, 1984). Participants were asked to report the extent to which they experienced distinct emotions over the past 24 hours on a scale from 0 (*not at all*) to 7 (*extremely*). The 12-item modified AAS includes positive (e.g., happy) and negative (e.g., frustrated) emotions, and notably, includes both high-arousal (e.g., joyful) and low-arousal (e.g., peaceful/serene) emotions. We also included two additional low-arousal emotion items (i.e., dull/bored, relaxed/calm), which were included in the negative and positive affect composites, respectively. The AAS demonstrated good reliability across all four timepoints (positive affect ranged from ω = 0.92 to 0.95; negative affect ranged from ω = 0.87 to 0.89).

##### State Self-Compassion

Participants reported their level of self-compassion during the negative event they described using the State Self-Compassion Scale Short Form (SSCS-S; (Neff et al., 2019). The SSCS-S comprises 6 items based on the SCS (Neff, 2003), phrased in the present tense to capture momentary feelings of self-compassion (rather than trait-like tendencies). The SSCS-S includes items that represent each of the three components of self-compassion, each with a positively-worded item (e.g., “I am giving myself the caring and tenderness I need”) and a negatively-worded item (e.g., “I feel intolerant and impatient toward myself”). Participants rated each item on a scale from 1 (*not at all true for me*) to 5 (*very true for me*). The SSCS-S demonstrated good reliability across all four timepoints (ω ranged from 0.83 to 0.86).

### Analytic Approach

We tested our preregistered hypotheses using two main approaches. For outcomes measured at pretest and posttest, we used residualized change analyses to detect condition differences. That is, we predicted outcomes at T_4_ (posttest) from condition, controlling for T_1_ (pretest) status. Experimental condition was dummy-coded such that the cognitive self-kindness condition served as the reference group for pairwise comparisons between groups. Thus, a negative coefficient would indicate that the cognitive self-kindness condition is higher than the group of interest for a particular outcome. Post-hoc contrast analyses were conducted using the emmeans R package with Tukey corrections to control the family-wise error rate across pairwise comparisons.

For outcomes measured at all four timepoints, we used multilevel growth models to detect condition differences. These models included fixed effects of experimental condition, time (centered at pretest), and their interaction (i.e., condition X time), along with random intercepts and slopes. Random slopes were only dropped from models due to nonconvergence. Multilevel models were estimated using full information maximum likelihood (FIML) to account for missing data. Finally, we tested a set of preregistered exploratory moderator variables in our multilevel models, including gender, ethnicity, first-generation status, and international student status.

## Results

### Does a Short, Self-Directed Self-Kindness Writing Exercise Improve Well-being?

Contrary to our hypothesis (Hypothesis 1) that participants in the cognitive self-kindness condition would report bigger increases in affective well-being and bigger decreases in brooding rumination and depression and anxiety symptoms relative to the control condition, our residualized change analyses did not detect significant differences for these outcomes (Table 2).

**Table 2:** Between-Condition Comparisons

	Hypotheses 1–3						Hypothesis 4		
	Cognitive Self-Kindness (0) v. Behavioral Self-Kindness (1)			Cognitive Self-Kindness (0) v. Control (1)			Control (0) v Both Kindness Conditions (1)		
Outcome	*β*	95% CI	*p*	*β*	95% CI	*p*	*β*	95% CI	*p*
Life Satisfact	0.05	-0.10, 0.20	0.515	-0.10	-0.26, 0.05	0.197	0.13	-0.01,0.27	0.062
Positive Aff.*	0.05	-0.02, 0.11	0.182	0.01	-0.05, 0.08	0.676	0.00	-0.05, 0.07	0.756
Negative Aff.*	0.00	-0.06, 0.08	0.804	-0.01	-0.08, 0.05	0.698	0.02	-0.04, 0.08	0.559
Brooding Rum	0.00	-0.12, 0.11	0.938	0.04	-0.08, 0.16	0.497	-0.06	-0.21, 0.09	0.406
Depression	-0.14	-0.30, 0.02	0.082	-0.13	-0.30, 0.03	0.110	0.06	-0.08, 0.20	0.405
Anxiety	-0.10	-0.26, 0.05	0.194	-0.09	-0.25, 0.07	0.263	0.04	-0.10, 0.18	0.590
Stress	-0.07	-0.23, 0.09	0.400	-0.11	-0.27, 0.05	0.200	0.07	-0.07, 0.22	0.308
Self-Comp	0.14	0.00, 0.27	**0.048**	-0.06	-0.20, 0.08	0.400	0.13	0.01, 0.25	**0.035**
Self-Esteem	-0.01	-0.15, 0.13	0.800	-0.10	-0.24, 0.05	0.200	0.09	-0.03, 0.21	0.155
Self-Worth	0.05	-0.12, 0.22	0.500	-0.01	-0.18, 0.17	0.950	0.03	-0.12, 0.18	0.678
Self-Efficacy	-0.08	-0.24, 0.08	0.300	-0.10	-0.26, 0.07	0.200	0.06	-0.09, 0.20	0.450

Life Satisfact. = Life Satisfaction. Positive Aff. = Positive Affect. Negative Aff. = Negative Affect. Brooding Rum. = Brooding Rumination. Each row represents a regression model. Models testing hypotheses 1–3 include a dummy-coded variable with cognitive self-kindness coded as the reference group. Models testing hypothesis 4 include a dummy-coded variable with the control condition coded as the reference group, compared to both self-kindness conditions (collapsed). All models represent residualized change models unless otherwise specified. Bolded text indicates statistically significant predictors (*p* < .05). *Indicates standardized coefficients for time by condition interactions in multilevel growth models for outcomes measured at all four timepoints

### Does the Type of Self-Kindness Exercise (Cognitive v. Behavioral) Impact Well-Being Outcomes?

Also contrary to our hypothesis (Hypothesis 2) that participants in the cognitive self-kindness condition would report greater increases in self-compassion and greater decreases in brooding rumination and depression and anxiety symptoms relative to those in the behavioral self-kindness condition, our residualized and multilevel models indicated no significant differences (Table 2).

The only significant difference among conditions for our pre-post outcomes was for trait self-compassion. Surprisingly, participants in the behavioral self-kindness condition reported greater trait self-compassion at posttest relative to those in the cognitive self-kindness condition (*β* = 0.14, CI_95%_ = [0.00, 0.27], *p* =.048). Ancillary analyses revealed that those in the behavioral self-kindness condition also reported significantly higher trait self-compassion at posttest relative to the control condition (*β* = 0.20, CI_95%_ = [0.06, 0.34], *p* =.005).

Our third hypothesis was also not supported. We expected that participants in the cognitive self-kindness condition would report greater self-esteem, self-efficacy, and self-worth relative to those in the behavioral self-kindness and control conditions, but our residualized change and multilevel models detected no such differences (Table 2). Supplementary analyses with pairwise comparisons between behavioral and self-kindness conditions are presented in Table S1 (Supplement).

Our multilevel models also did not support Hypothesis 4. Despite our expectation that our experimental conditions (collapsed) would report greater state self-compassion (Table 3) and daily positive affect (Table 4) and lower daily negative affect (Table 5) relative to the control condition, our multilevel models detected no such effects.

**Table 3:** Multilevel growth models predicting state self-compassion: self-kindness conditions vs. control

		Unconditional Growth			Kindness Conditions vs. Control		
		Estimate	95% CI	*p*	Estimate	95% CI	*p*
Fixed Effects	Intercept	−0.12	−0.19, −0.05	**0.001**	−0.13	−0.26, −0.01	**0.029**
	Time	0.09	0.06, 0.11	** < 0.001**	0.11	0.06, 0.15	** < 0.001**
	Behavioral Self-Kindness				0.04	−0.13, 0.21	0.607
	Cognitive Self-Kindness				0.01	−0.16, 0.18	0.916
	Time*Behavioral Self-Kindness				−0.02	−0.09, 0.04	0.467
	Time*Cognitive Self-Kindness				−0.03	−0.10, 0.03	0.337
Random Effects	Level 1: σ^2^	0.36			0.36		
	Level 2 Intercept: τ_00_	0.65			0.65		
	Level 2 Slope: τ_11_	0.03			0.03		
	ICC	0.63			0.63		
	N	734			734		
	Observations	2332			2332		

All variables are standardized. The time parameter was centered at baseline. *p-*values for fixed effects were calculated using the Satterthwaite approximation via the lmerTest package in R. Bolded text indicates statistically significant predictors (*p* <.05)

**Table 4:** Multilevel growth models predicting daily positive affect: self-kindness conditions vs. control

		Unconditional Growth			Kindness Conditions vs. Control		
		Estimate	95% CI	*p*	Estimate	95% CI	*p*
Fixed Effects	Intercept	0.00	−0.07, 0.06	0.908	−0.02	−0.13, 0.09	0.703
	Time	0.01	−0.02, 0.04	0.476	0.00	−0.04, 0.05	0.885
	Behavioral Self-Kindness				0.10	−0.06, 0.26	0.211
	Cognitive Self-Kindness				−0.05	−0.21, 0.11	0.546
	Time*Behavioral Self-Kindness				0.03	−0.04, 0.10	0.368
	Time*Cognitive Self-Kindness				−0.01	−0.08, 0.05	0.676
Random Effects	Level 1: σ^2^	0.38			0.38		
	Level 2 Intercept: τ_00_	0.51			0.50		
	Level 2 Slope: τ_11_	0.03			0.03		
	ICC	0.62			0.61		
	N	738			738		
	Observations	2345			2345		

All variables are standardized. The time parameter was centered at baseline. *p-*values for fixed effects were calculated using the Satterthwaite approximation via the lmerTest package in R. Bolded text indicates statistically significant predictors (*p* <.05)

**Table 5:** Multilevel growth models predicting daily negative affect: self-kindness conditions vs. control

		Unconditional Growth			Kindness Conditions vs. Control		
		Estimate	95% CI	*p*	Estimate	95% CI	*p*
Fixed Effects	Intercept	0.11	0.04, 0.18	**0.002**	0.14	0.04, 0.27	**0.02**
	Time	−0.07	−0.10, −0.04	** < 0.001**	−0.08	−0.12, −0.02	**0.001**
	Behavioral Self-Kindness				−0.11	−0.29, 0.04	0.195
	Cognitive Self-Kindness				0.01	−0.18, 0.16	0.878
	Time*Behavioral Self-Kindness				0.02	−0.06, 0.08	0.521
	Time*Cognitive Self-Kindness				0.01	−0.08, 0.05	0.698
Random Effects	Level 1: σ^2^	0.39			0.39		
	Level 2 Intercept: τ_00_	0.60			0.60		
	Level 2 Slope: τ_11_	0.03			0.03		
	ICC	0.60			0.60		
	N	738			738		
	Observations	2345			2345		

All variables are standardized. The time parameter was centered at baseline. *p-*values for fixed effects were calculated using the Satterthwaite approximation via the lmerTest package in R. Bolded text indicates statistically significant predictors (*p* <.05)

### Exploratory Aim 1: Test Individual Difference Moderators on Intervention Effects

Although the hypothesized between-condition differences were largely null, moderation analyses revealed that outcomes varied based on several individual difference variables. While gender and international student status were not significant moderators of intervention effects, racial-ethnic identity (Asian and Latinx identity, separately) and first-generation college status emerged as significant moderators, as detailed below. Supplementary analyses examining demographic differences in self-reported intervention difficulty and effort are presented in Tables S4-S8. Asian participants constituted 45.87% of our sample, while those of Hispanic/Latinx background represented 29.91% of our sample. First-generation college students were also well-represented in our sample and constituted nearly half of participants (49.17%).

#### Asian Participants

Asian identity modified condition effects for the following outcomes:

##### Self-Esteem

**Fig. 1:**
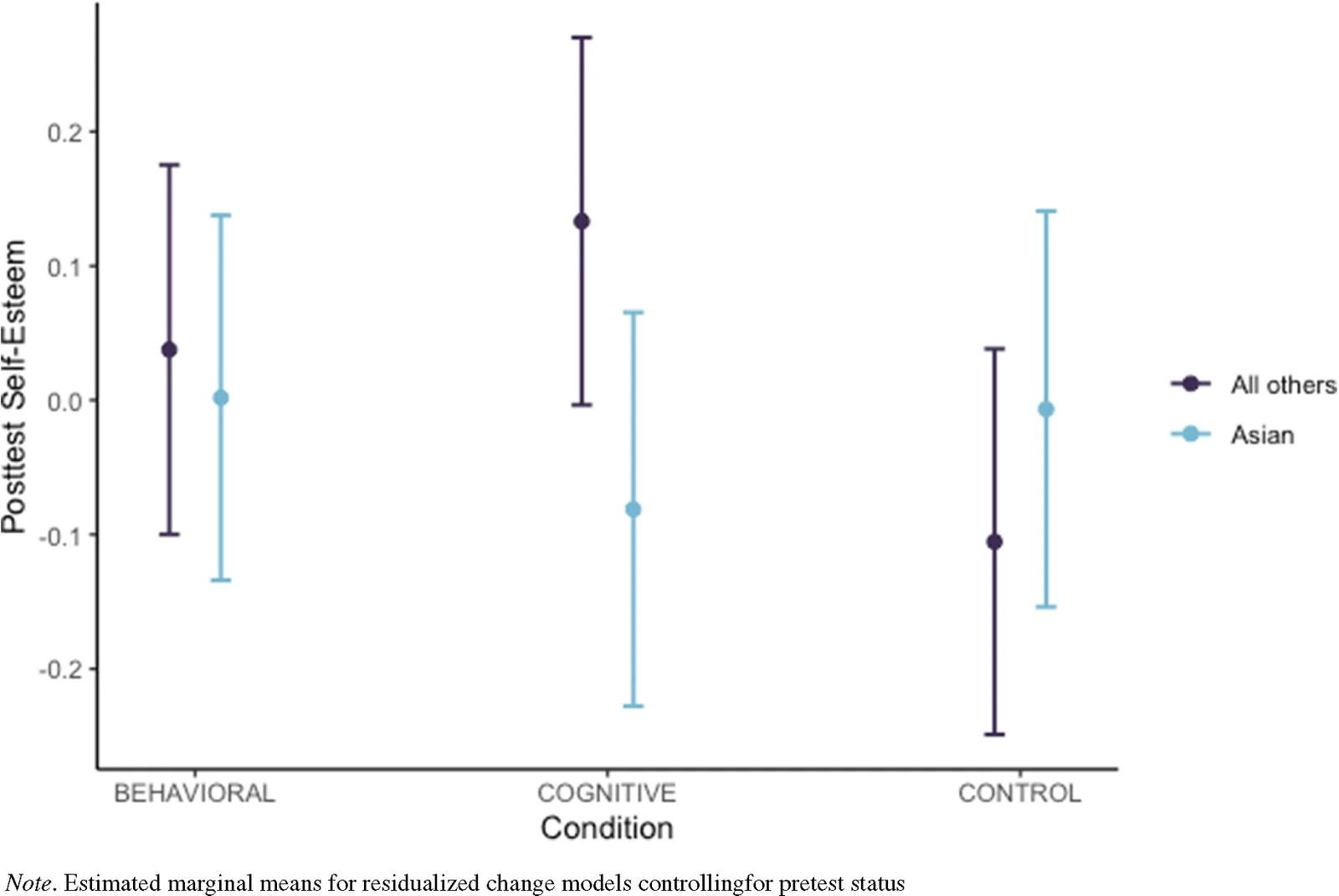
Differences in Self-Esteem by Asian Identity and Condition

Residualized change models predicting posttest self-esteem revealed a significant interaction between condition and Asian identity (*β* = 0.31, CI_95%_ = [0.02, 0.60], *p* =.034; see Fig. 1). Post hoc contrasts revealed that those who did not identify as Asian reported higher self-esteem in the cognitive self-kindness condition than in the control condition (estimated mean difference = 0.24, CI_95%_ = [0, 0.48, 0], *t*(466) = 2.35, *p* =.049), while Asian participants in the cognitive self-kindness condition reported lower self-esteem than all other participants in the cognitive self-kindness condition (estimated mean difference = −0.21, CI_95%_ = [−0.42, −0.01], *t*(466) = −2.09, *p* =.037).

##### Self-worth

Residualized change models predicting posttest self-worth revealed a significant interaction between the condition and Asian identity (*β* = 0.38, CI_95%_ = [0.04, 0.73], *p* =.030), such that the difference between the control and cognitive condition depended on whether participants were Asian relative to other ethnicities. Post hoc contrasts did not reveal any significant pairwise differences, however (all *p*-values >.05). Post hoc results for these analyses are presented in Table S2 (Supplement).

#### Latinx Participants

With Latinx participants constituting almost a third of our sample, Latinx identity moderated condition effects for the following outcomes:

##### Life Satisfaction

**Fig. 2:**
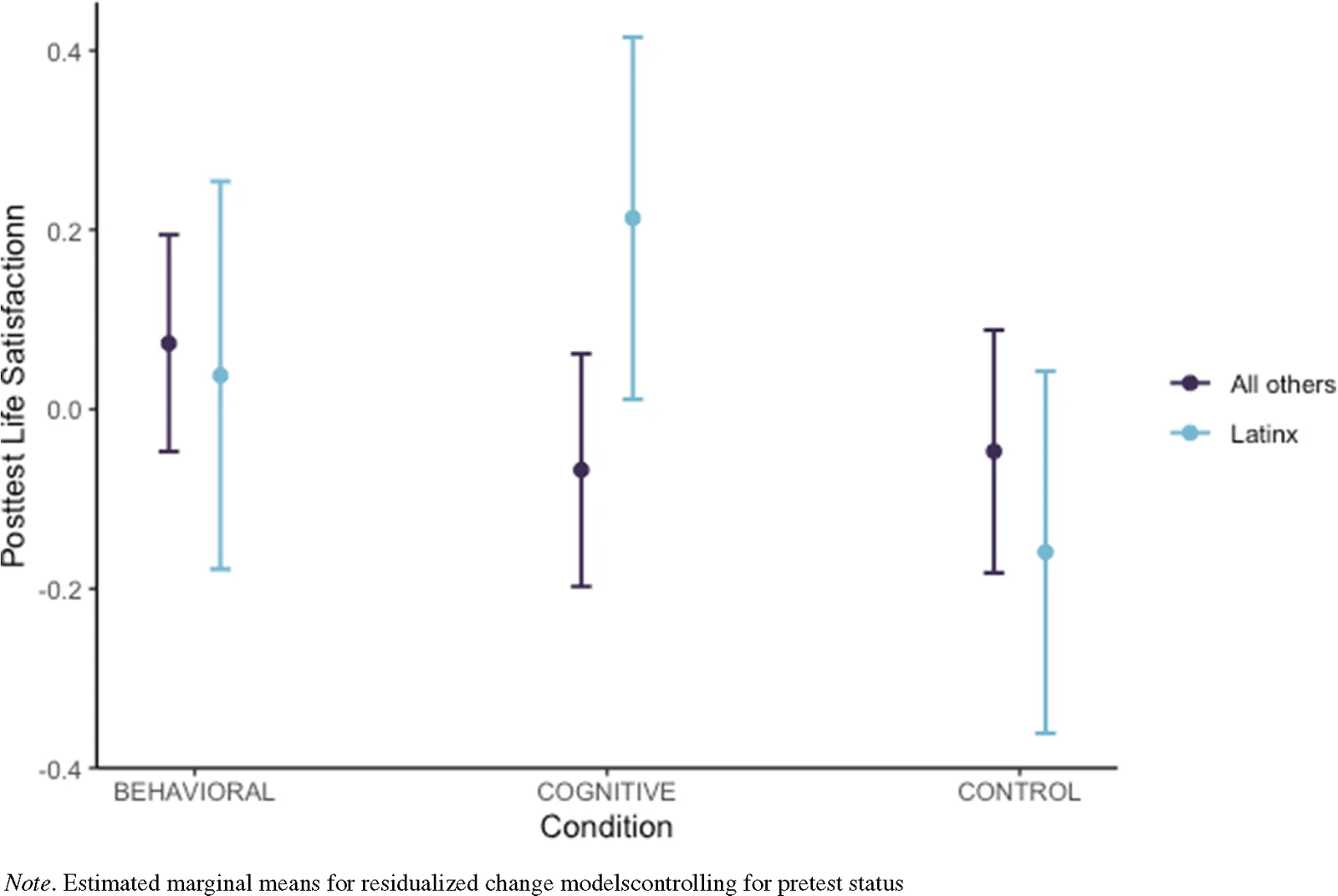
Differences in Life Satisfaction by Latinx Identity and Condition

Residualized change models predicting posttest life satisfaction revealed a significant interaction between condition and Latinx identity (*β* = −0.39, CI_95%_ = [−0.74, −0.05], *p* =.025; see Fig. 2). Post-hoc contrasts revealed that Latinx participants in the cognitive self-kindness condition reported greater posttest life satisfaction relative to Latinx participants in the control condition (estimated mean difference = 0.37, CI_95%_ = [0.03, 0.72], *t*(466) = 2.55,* p* =.03), and that Latinx participants in the cognitive condition reported greater posttest life satisfaction relative to all other participants in the cognitive condition (estimated mean difference = 0.28, CI_95%_ = [0.04, 0.52], *t*(466) = 2.29*, p* =.02).

##### Self-Esteem

**Fig. 3:**
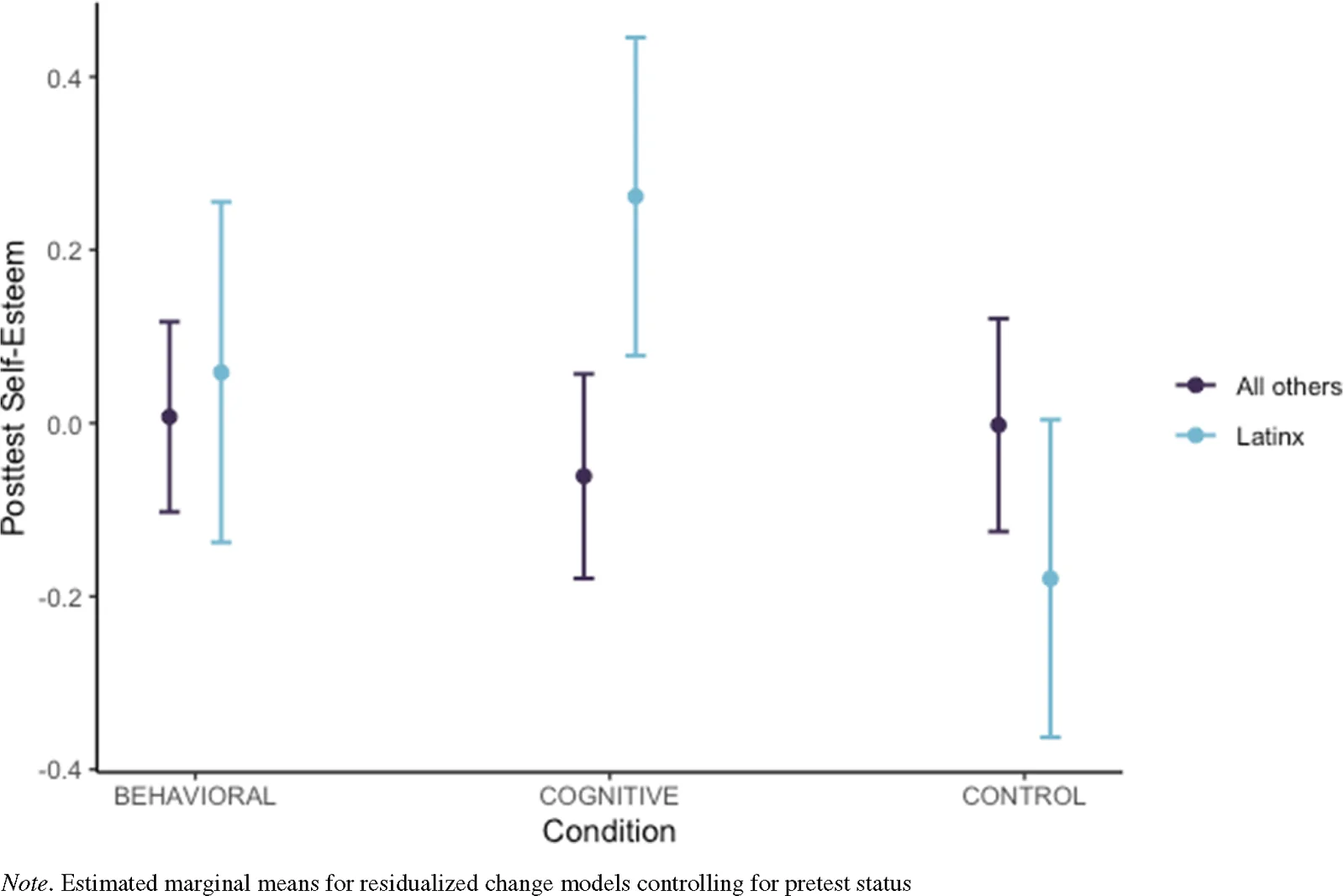
Differences in Self-Esteem by Latinx Identity and Condition

Residualized change models predicting posttest self-esteem revealed a significant interaction between condition and Latinx identity (*β* = −0.50, CI_95%_ = [−0.81, −0.19], *p* =.002; see Fig. 3. Post-hoc contrasts paralleled the analyses for life satisfaction: Latinx participants in the cognitive self-kindness condition reported significantly higher self-esteem at posttest relative to Latinx participants in the control condition (estimated mean difference = 0.44, CI_95%_ = [−0.75, −0.13], *t*(466) = −3.33*, p* =.003); and Latinx participants in the cognitive self-kindness condition reported significantly higher self-esteem than all other participants in the cognitive self-kindness condition (estimated mean difference = 0.32, CI_95%_ = 0.10, 0.54], *t*(466) = 2.90*, p* =.004).

##### Self-Worth

**Fig. 4:**
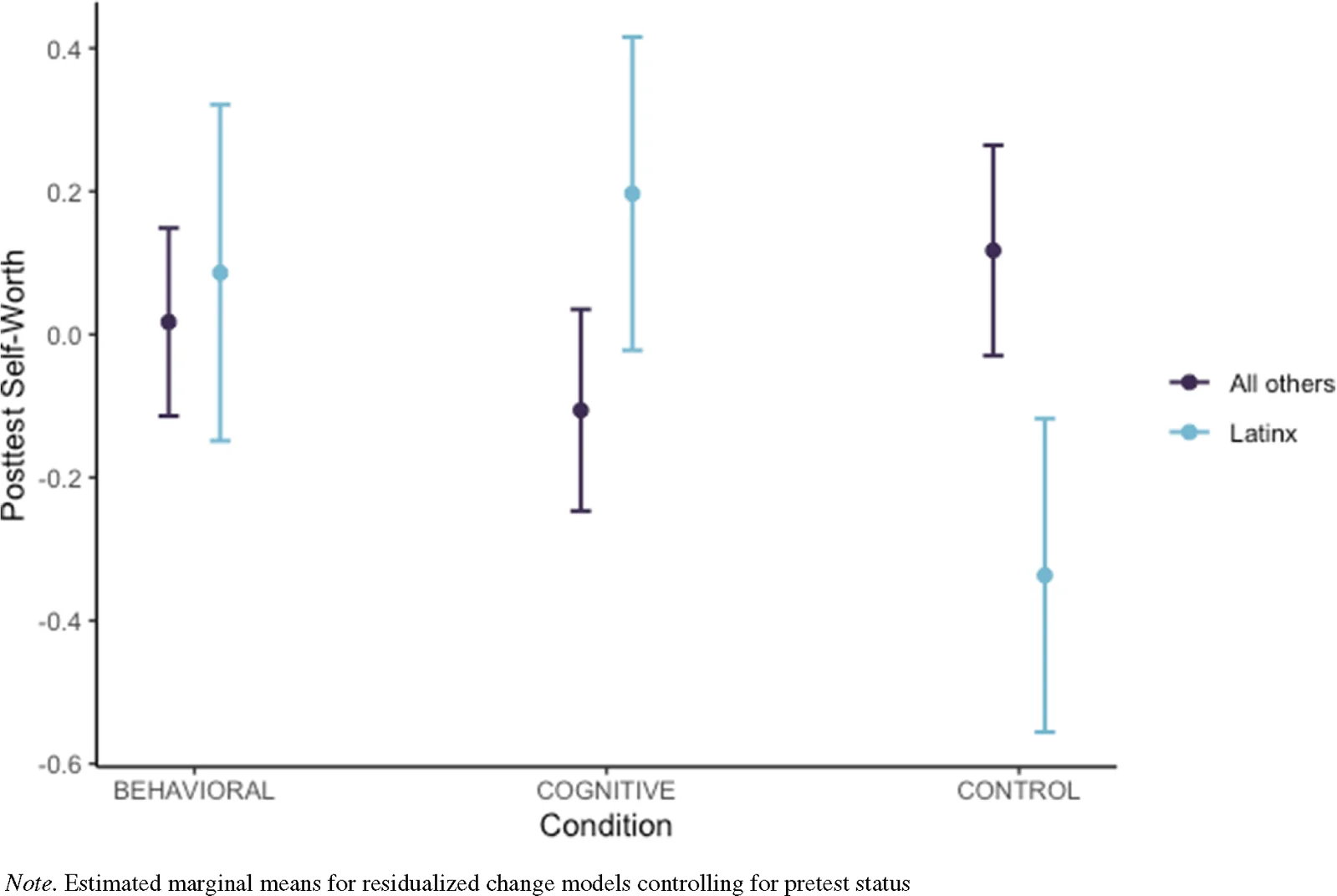
Differences in Self-Worth by Latinx Identity and Condition

Residualized change models predicting posttest self-worth again revealed a significant interaction between condition and Latinx identity (*β* = −0.76, CI_95%_ = [−1.1, −0.39], *p* <.001; see Fig. 4). Post-hoc contrasts showed that Latinx participants in the cognitive self-kindness condition reported significantly higher self-worth at posttest relative to Latinx participants in the control condition (estimated mean difference = 0.53, CI_95%_ = [−0.91, −0.16], *t*(466) = −3.38*, p* =.002); and Latinx participants in the cognitive self-kindness condition reported significantly higher self-worth than all other participants (estimated mean difference = 0.30, CI_95%_ = [0.04, 0.56], *t*(466) = 2.28*, p* =.02). Finally, Latinx participants in the control condition also reported lower self-worth than all other participants (estimated mean difference = −0.45, CI_95%_ = [−0.72, −0.19], *t*(466) = −3.37*, p* =.001). Although the interaction between the behavioral self-kindness condition and ethnicity did not reach statistical significance, post-hoc contrasts indicated that Latinx participants in the behavioral self-kindness group reported higher self-worth than Latinx participants in the control group (estimated mean difference = 0.42, CI_95%_ = [−0.81, −0.04], *t*(466) = −2.58*, p* =.027).

#### First-Generation College Students

With first-generation college students compromising nearly half of our sample, first-generation college status moderated the following outcomes:

##### Life Satisfaction

**Fig. 5:**
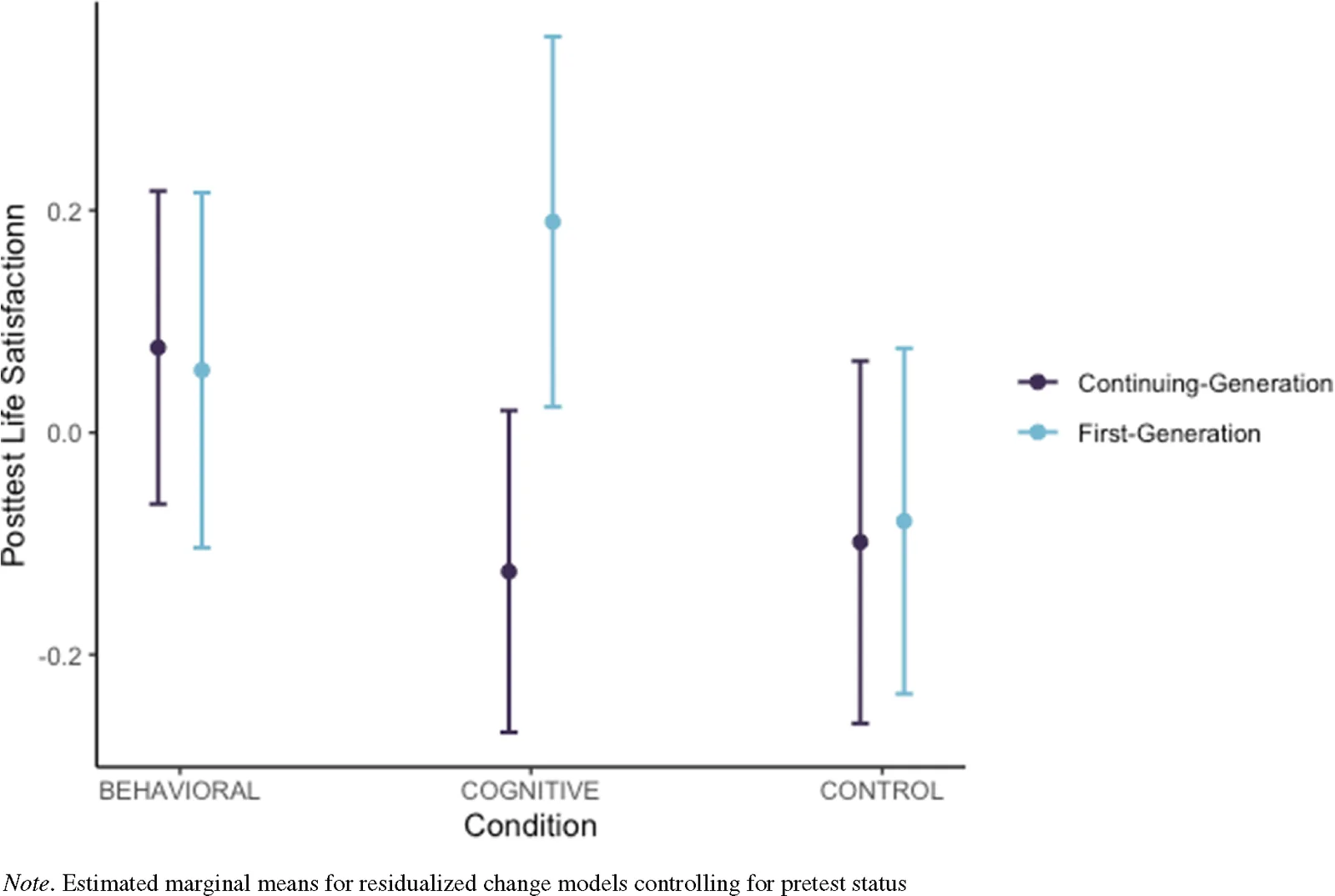
Differences in Life Satisfaction by First-Generation Status and Condition

Residualized change models predicting posttest life satisfaction revealed a significant interaction between condition and first-generation college status (*β* = −0.34, CI_95%_ = [−0.64, −0.03], *p* =.03; see Fig. 5). Post-hoc contrasts did not detect statistically significant pairwise condition differences, but did reveal that first-generation college students in the cognitive self-kindness condition reported greater posttest life satisfaction relative to continuing-generation college students in the same condition (estimated mean difference = 0.32, CI_95%_ = [0.09, 0.53], *t*(463) = 2.80, *p* =.005).

##### Self-Efficacy

**Fig. 6:**
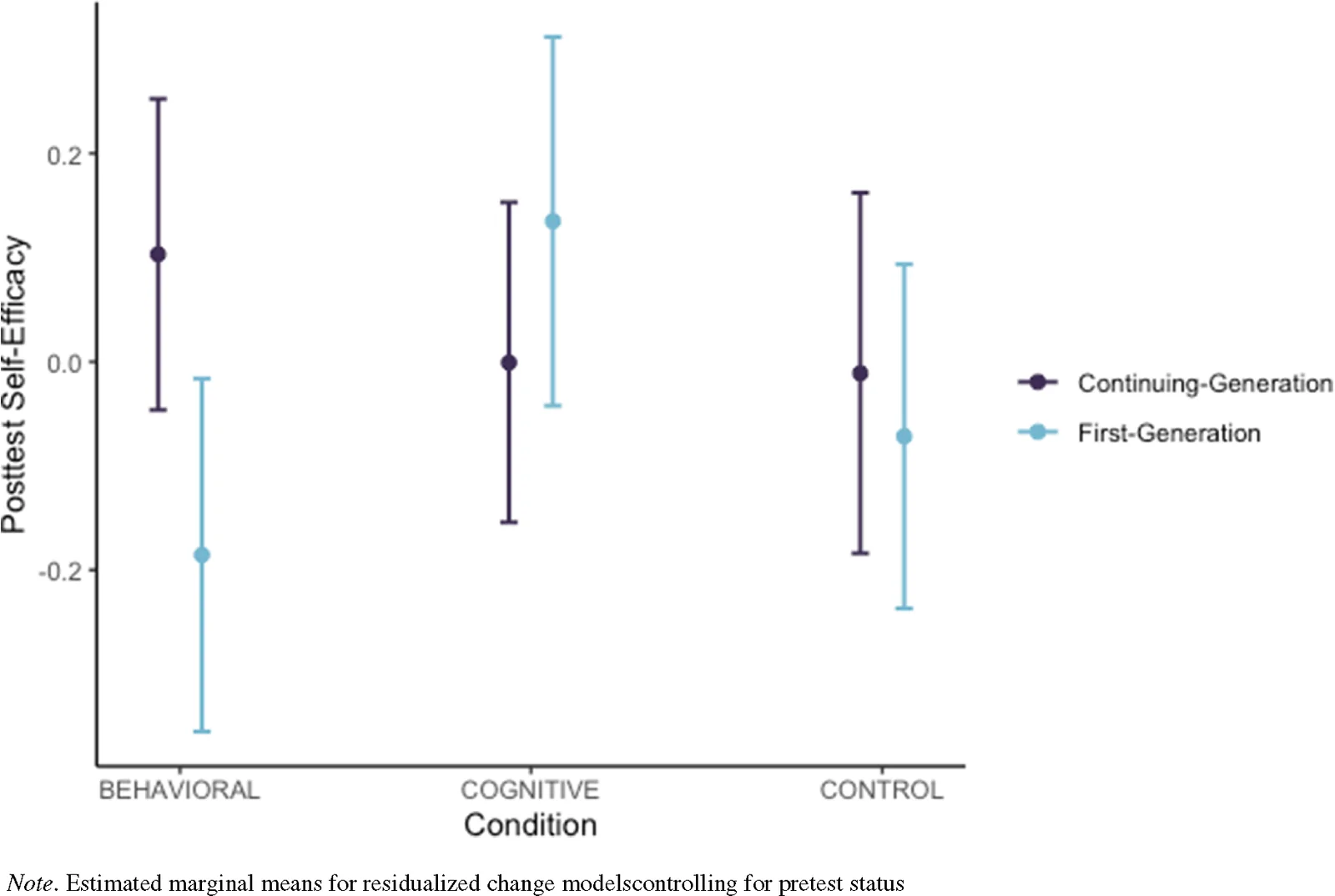
Differences in Self-Efficacy by First-Generation Status and Condition

Residualized change models predicting posttest self-efficacy revealed a significant interaction between condition and first-generation college status (*β* = −0.42, CI_95%_ = [−0.75, −0.10], *p* =.011; see Fig. 6). Post-hoc contrasts indicated that first-generation college students in the cognitive self-kindness condition report higher self-efficacy at posttest relative to first-generation college students in the control condition (estimated mean difference = 0.32, CI_95%_ = [0.03, 0.62], *t*(463) = 2.56, *p* =.029), and that first-generation college students in the behavioral self-kindness condition report lower self-efficacy than continuing-generation college students in the same condition (estimated mean difference = −0.28, CI_95%_ = [−0.52, −0.06], *t*(463) = −2.5, *p* =.013).

##### Brooding Rumination

Residualized change models predicting posttest brooding rumination revealed a significant interaction between condition and first-generation college status (*β* = −0.37, CI_95%_ = [−0.71, −0.02], *p* =.037), indicating that the difference between the control and cognitive self-kindness conditions varied based on whether students were the first in their family to attend college. Post hoc contrasts did not reveal any significant pairwise differences, however (all *p*-values >.05). Post hoc results for these analyses are presented in Table S3 (Supplement).

## Discussion

In our 1-week experimental study, we examined whether seeing oneself from the perspective of a kind friend (cognitive self-kindness) and performing kind acts for oneself (like enjoying a favorite meal; behavioral self-kindness) differentially impact the development of self-compassion and well-being. Furthermore, we explored whether individual and cultural differences moderate these intervention effects. Our study yielded some unexpected findings regarding the relative benefits of cognitive v. behavioral self-kindness and highlighted the role of individual differences in shaping self-compassion intervention outcomes.

### Behavioral Strategies as a Less Effortful Path to Self-Compassion?

Contrary to our expectations, participants who practiced cognitive self-kindness did not report greater increases in self-compassion and positive psychological outcomes relative to those in the behavioral self-kindness and control conditions. Another unexpected finding was that the *behavioral* self-kindness group, who were asked to perform kind acts for themselves, reported the highest levels of self-compassion at posttest relative to the other groups. These findings raise the possibility that acting kindly toward oneself could be another means to develop self-compassion relative to more firmly established cognitive (reflective) means (e.g., Gilbert, 2014; Neff & Germer, 2013).

To our knowledge, cognitive and behavioral self-kind exercises have not been directly compared before. Accordingly, our intervention is the first to suggest that encouraging behavioral acts of self-kindness may be more effective in promoting self-compassion. Notably, ancillary analyses indicated that our participants reported significantly less effort and marginally less difficulty carrying out the behavioral self-kindness condition relative to the cognitive self-kindness condition (see Supplement). If these findings replicate, encouraging participants to engage in behavioral self-kindness could be a more feasible intervention target than cognitive self-kindness, which may be more difficult to successfully carry out. Participants might find it easier to *treat* themselves with kindness through tangible actions, and such acts of self-care may enable them to *think* of themselves with more kindness over time.

Insights from other psychological research traditions suggest that “doing” rather than “thinking” is an easier entry point to improve mood and overall life adjustment. Depression researchers, for example, tout the benefits of behavioral activation—or, incorporating enjoyable events in daily routines, like taking like talking a walk or spending time with family (Jacobson et al., 2001)— for downstream psychological well-being. Behavioral activation is thought to break up a person’s limited behavioral routines and thinking that might trap them in a negative mood (Jacobson et al., 2001). Although cognitively focused solutions to depression might focus on working “inside-out” (i.e., challenging unhelpful thoughts), a combined cognitive-behavioral approach focuses on working “outside-in” (i.e., changing behavior first, so changes in thoughts can follow; Jacobson et al., 2001). In the uphill battle of depression, behaviors might be relatively easier to tackle before updating a longstanding way of thinking. In parallel, updating one’s enduring relationship with oneself might also be facilitated by self-kind behaviors first to allow downstream changes to self-appraisal. Additional research could explore and extend insights related to cognitive vs. behavioral-facilitated changes to self-compassion.

### Racial-Ethnic Identity and First-Generation College Status Impact Intervention Effects

In addition to our findings regarding the comparative efficacy of behavioral and cognitive self-kindness, preregistered moderation analyses revealed several ways that cultural and demographic differences might bear on intervention success. We found, for instance, that Asian participants in the cognitive self-kindness condition reported lower self-esteem relative to non-Asians. Further, Latinx and first-generation college participants appeared to have uniquely benefited from thinking kindly about themselves with respect to life satisfaction and several measures reflecting positive self-regard. There are three key implications of these moderation findings.

First, our finding that Asian participants reported significantly lower self-esteem relative to non-Asians within the cognitive self-kindness condition parallels other research that indicates that Asians, compared to non-Asians, tend to endorse holding both positive and negative aspects of self-regard at once (e.g., self-kindness simultaneously with over-identification with failure; Birkett, 2014). Accordingly, it is possible that asking Asian participants in our study to be kinder to themselves also unintentionally promoted negative self-reflection in them.

By contrast, supplementary analyses (see Tables S7 and S8 in Supplement) also indicate a main effect of Asian identity on self-reported effort in our intervention, such that Asian participants reported exerting less effort in our intervention (regardless of condition) relative to non-Asians. The relatively lower effort exerted by Asian participants might also reflect the well-replicated finding that Asians, relative to Westerners, engage in less self-enhancement (attempts to view and present oneself more favorably relative to others; Heine & Hamamura, 2007). With this literature in mind, it is plausible that both of our self-kindness conditions did not align with Asian cultural preferences related to the self (e.g., the instructions could be interpreted as self-indulgent), and were, therefore, unmotivating. Additional research is needed to tease apart the motivating mechanisms and efficacy of self-compassion interventions among Asian Americans.

Second, the benefits of cognitive self-kindness among our Latinx participants are consistent with some evidence pointing to the efficacy of self-compassion interventions for Latinx populations (e.g., Castellanos et al., 2020; Edwards et al., 2014). Our findings also echo cross-sectional findings that indicate Latinx individuals with greater self-compassion are more resilient against stressors related to acculturation (adjustment to a new culture; Piña-Watson et al., 2025) and the COVID-19 pandemic (Kroshus et al., 2023). It may be that thinking of oneself kindly amid external stressors relatively beyond personal control (e.g., cultural acceptance by others, limits to social and behavioral coping strategies during the COVID-19 pandemic, etc.) enables Latinx people to protect or bolster their well-being. The present study adds experimental evidence to the body of limited literature speaking to the benefits of self-compassion among Latinx individuals and offers a foundation for future mechanistic studies. With additional replication efforts, the continued development of self-compassion interventions tailored for Latinx populations may be fruitful.

Third, our preliminary results suggest that cognitive self-compassion may especially bolster the well-being of first-generation college students. First-generation college students face unique stressors in their educational journeys, including less informational and parental support, chronic stress, academic anxiety, financial difficulties, and competing responsibilities (House et al., 2020; Kroshus et al., 2021; Sy et al., 2011). Little to no research, to our acknowledge, addresses the prospective benefits of self-compassion for first-generation college students, and if such effects are more firmly established, self-compassion interventions present a promising avenue to address unique challenges among this population.

Among first-generation college students specifically, it may be useful to consider whether financial barriers contributed to the relative benefits of cognitive self-kindness (a cost-free exercise) over behavioral self-kindness (which could incur some costs related to hobbies, food, etc.). Relative to continuing-generation students, first-generation students tend to report more financial strain (Rehr et al., 2022). However, we did not collect financial demographics among our sample to test whether financial resources accounted for any between-condition variation in our outcomes. Supplementary analyses (see Tables S4-S7 in Supplement) indicate that condition did not significantly interact with demographic group (including first-generation status) to predict self-reported difficulty and effort. A significant main effect of first-generation status did indicate that first-generation students, relative to continuing-generation students, reported more effort, but not difficulty, in our intervention (regardless of condition). Plausibly, the fact that first-generation students reported more effort but not difficulty in our intervention might reflect greater motivation to engage in self-kindness. Alternatively, it is also possible that greater chronic stressors and financial self-reliance (Rehr et al., 2022; Sy et al., 2011) among first-generation students make it more effortful to exercise either behavioral or cognitive self-kindness. It is also possible that greater stressors among first-generation students also compromise their capacity (cognitive, financial, and physical) to engage in self-kindness. Future investigations could collect and examine data on the financial resources and other barriers/supports to self-kindness among first-generation college students.

Notably, a majority of first-generation students were also Latinx, and nearly all Latinx students reported being first-generation college students. Thus, it is not possible to fully disentangle the effects of being a Latinx or first-generation college student in the present research, as removing Latinx participants from the first-generation analyses would create subgroups that are too small to detect significant differences. Additional research is needed to replicate the present results and extend this work to better understand how multiple identities may influence the efficacy of self-kindness interventions.

## Limitations

A number of study limitations should be acknowledged. First, our short, 1-week intervention may not have been long enough for participants to reap benefits (Ferrari et al., 2019). Although other self-compassion interventions as short as 3 minutes have been effective (e.g., Gobin et al., 2022), we did not observe most of our intended intervention effects. Second, our two experimental conditions focused on targeting one component of self-compassion (self-kindness) and overlooked the components of mindfulness and common humanity. A multimodal intervention targeting all three components of self-compassion may be more efficacious (e.g., Neff & Germer, 2013). Future research could test each of the three components separately to determine their unique proximal mechanisms, as well as test all three components together to deliver a more powerful intervention. Third, we did not administer a manipulation check to assess whether participants in the behavioral and cognitive self-kindness conditions engaged in acts of self-kindness not assigned to them (i.e., whether participants in the behavioral self-kindness condition also engaged in cognitive self-kindness and vice versa). Future researchers could account for such potential spillover effects across self-kindness conditions by inquiring about and examining the nature of self-kindness exercised as part of the intervention.

## Summary

In our 1-week self-compassion intervention, undergraduates prompted to perform acts of kindness for themselves reported greater self-compassion at posttest than students prompted to think about themselves kindly. These results suggest that encouraging people to treat themselves kindly could be an effective and relatively more effortless way to bolster self-compassion in the short-term, relative to more cognitive strategies, which likely require more practice and time. Notably, our findings also suggest that thinking of oneself kindly may be uniquely beneficial to the well-being of Latinx and first-generation college students, paving the way for further applied research to address their unique challenges and bolster their resilience.

## Supplementary Information

Below is the link to the electronic supplementary material.

Supplementary file1 (DOCX 37 KB)

## Additional Information

### Data Availability

Data and analytic code are available from the corresponding author upon request.
