# Supplementary material for: Pathways to Self-Compassion: The Effects of Self-Kind Behaviors and Self-Kind Thoughts on Self-Compassion and Well-Being
Source: Affect Sci. 2026 Jun 24;7(3):447–63. doi: 10.1007/s42761-026-00379-4 (PMC13582781; doi:10.1007/s42761-026-00379-4)
Supplement: Supplementary file 1 [file 42761_2026_379_MOESM1_ESM.docx]

**Supplement**

**Condition Instructions**

***Cognitive Self-Kindness***

Now, we would like you to complete a brief writing exercise about responding to stressful situations.

1. First, think about times when a close friend of yours feels really bad about themselves or is struggling in some way. How would you respond to your friend in this situation (especially when you’re at your best)?
2. Please write about what you typically do, what you say, and note the tone in which you typically talk to your friends when they are going through a hard time.
3. Now think about times when you feel bad about yourself or are struggling. How do you typically respond to yourself in these situations? Please write down what you typically do, what you say or think to yourself, and note the tone in which you talk to yourself.
4. Did you notice a difference between the way you typically talk to your friends and the way you typically respond to yourself in difficult situations? If so, ask yourself why. What factors or fears come into play that lead you to treat yourself and others so differently?
5. Please write down how you think things might change for you if you responded to yourself in the same way you typically respond to a close friend when you’re suffering. Remember, it is the human condition to be imperfect, and feelings of suffering, failure, and inadequacy are part of the experience of living a human life.
   Why not try treating yourself like a good friend and see what happens?

***Behavioral Self-Kindness***

Now, we would like you to complete a brief exercise about responding to stressful situations. Take a moment to remember the stressful or difficult experience you just described.

We often neglect to do nice things for ourselves to make us feel better during difficult times. Tomorrow, please perform an act of kindness for yourself for at least 15 minutes. You can choose to do whatever you’d like, but make sure you do choose something out of the ordinary that you do for yourself with a little extra effort.

Examples include having your favorite meal, treating yourself to a massage, or spending time on your favorite hobby. These nice things for yourself do not need to be the same as the examples listed above, and although they may involve other people, they should be things that you do explicitly for yourself, not others.

Please list three things you might do to make yourself feel better tomorrow:

1. ____
2. ____
3. ____

***Active Control***

One way to reduce stress is to keep track of how you spend your time. Doing this will help you organize your time better and help you feel less overwhelmed.

Tomorrow, as you go about your day, please keep track of your activities. You do not need to remember who you are with or how you are feeling during that time. Instead, just try to remember factual information about what you are doing. Do not alter your routine in any way; simply keep track of what you do.

When you log back into the study, you will be asked to write an outline of what you did. For example: Morning: ate breakfast, went to work, ate lunch with coworkers. Afternoon: started a new project, held a meeting, went to the gym. Evening: ate dinner, watched TV, went to bed. Only the facts are important.

**Supplementary Measure**

***Activity Effort and Difficulty***

Participants also completed a single item to assess how much effort they put into their assigned activity, rating their effort from 1 (*not a lot of effort*) to 7 (*a great deal of effort*) and difficulty of the activity from 1 (*not difficult at all*) to 7 (*very difficult*) at the beginning of the T_2_ through T_4_ assessments. The three measurements of effort were averaged into a single composite for each participant, as were the three measurements of difficulty.

**Supplementary Results**

A one-way ANOVA identified significant differences in terms of the amount of effort participants put into their assigned activities throughout the intervention period (*F*[2, 600] = 34.35, p < .001). Specifically, post-hoc Tukey HSD tests revealed that participants in the control condition (M = 4.01, SD = 1.29) reported putting less effort into their activities relative to the behavioral self-kindness condition (M = 4.50, SD = 1.28; *p* < .001) and the cognitive self-kindness condition (M = 5.07, SD = 1.23; *p* < .001). Participants in the cognitive self-kindness condition reported putting more effort into their assigned activity relative to the behavioral self-kindness condition (*p* < .001). Similarly, conditions differed in terms of how difficult participants found them, although these differences were only marginally significant (*F*[2, 600] = 2.649, *p =* .072).

**Table S1**

Control v. Behavioral Condition Comparisons

| Outcome | *β* | 95% CI | *p* |
| --- | --- | --- | --- |
| Life Satisfaction | 0.15 | 0.00, 0.31 | 0.051 |
| Positive Affect* | 0.03 | -0.04, 0.10 | 0.368 |
| Negative Affect* | 0.02 | -0.05, 0.09 | 0.521 |
| Brooding Rumination | -0.05 | -0.17, 0.07 | 0.443 |
| Depression | -0.01 | -0.17, 0.15 | 0.923 |
| Anxiety | -0.01 | -0.17, 0.14 | 0.877 |
| Stress | 0.04 | -0.12, 0.20 | 0.626 |
| Self-Compassion | 0.20 | 0.06, 0.34 | **0.005** |
| Self-Esteem | 0.08 | -0.06, 0.23 | 0.246 |
| Self-Worth | 0.06 | -0.11, 0.23 | 0.516 |
| Self-Efficacy | 0.02 | -0.15, 0.18 | 0.847 |

*Note.* Each row represents a regression model. Models include a dummy-coded variable with control condition coded as the reference group. All models represent residualized change models unless otherwise specified. Bolded text indicates statistically significant predictors (*p* < .05). *Indicates standardized coefficients for time by condition interactions in multilevel growth models for outcomes measured at all four timepoints.

**Table S2**

Tukey Honestly Significant Difference (HSD) Post-Hoc Tests: Asian Identity and Condition Effects on Self-Worth (Standardized)

| Condition | Est. Mean  Difference  (Asian – Non-Asian) | SE | *df* | 95% CI | *t* | *p* |
| --- | --- | --- | --- | --- | --- | --- |
| Cognitive Self-Kindness | -0.208 | 0.123 | 466 | -0.46, 0.03 | -1.695 | 0.091 |
| Behavioral Self-Kindness | -0.151 | 0.118 | 466 | -0.38, 0.08 | -1.280 | 0.201 |
| Control | 0.175 | 0.126 | 466 | -0.07, 0.42 | 1.386 | 0.166 |

*Note.* Est. mean difference = estimated mean difference. SE = Standard error. Estimated mean difference in self-worth between Asian – Non-Asian subgroups with negative mean difference indicating lower self-worth amongst Asians relative to non-Asians.

**Table S3**

Tukey Honestly Significant Difference (HSD) Post-Hoc Tests: First-Generation Status and Condition Effects on Brooding Rumination (Standardized)

| Condition | Est. Mean  Difference  (First. Gen.–Cont. Gen.) | SE | *df* | 95% CI | *t* | *p* |
| --- | --- | --- | --- | --- | --- | --- |
| Cognitive Self-Kindness | 0.172 | 0.123 | 462 | -0.07, 0.41 | 1.399 | 0.162 |
| Behavioral Self-Kindness | -0.133 | 0.120 | 462 | -0.37, 0.10 | -1.111 | 0.267 |
| Control | -0.195 | 0.126 | 462 | -0.44, 0.05 | -1.554 | 0.121 |

*Note.* Est. mean difference = estimated mean difference. First. Gen. = first-generation status. Cont. Gen. = continuing-generation status. SE = Standard error. Estimated mean difference in brooding rumination between first-generation and continuing-generation student subgroups with negative mean difference indicating lower brooding rumination amongst first-generation students relative to continuing-generation students.

**Table S4**

*Average Difficulty (Standardized) by Condition and Demographic Group*

| Demographic Group | Behavioral  Self-Kindness | | Cognitive  Self-Kindness | | Control |  |
| --- | --- | --- | --- | --- | --- | --- |
|  | M(SD) | | | | |  |
| Asian | -0.12 (0.90) | 0.04 (0.91) | | -0.05 (1.03) | | |
| Non-Asian | -0.11 (0.96) | 0.17 (0.96) | | -0.07 (0.94) | | |
| Latinx | -0.06 (1.06) | 0.11 (0.90) | | -0.13 (0.90) | | |
| Non-Latinx | -0.13 (0.89) | 0.11 (0.95) | | -0.02 (1.01) | | |
| First-Generation | -0.14 (1.01) | 0.06 (0.97) | | -0.14 (0.90) | | |
| Continuing-Generation | -0.12 (0.82) | 0.15 (0.92) | | 0.03 (1.07) | | |

**Table S5**

*Two-Way ANOVA of Effects of Condition and Demographic Group on Difficulty (Standardized)*

|  | *df* | SS | MS | *F* | *p* |  |
| --- | --- | --- | --- | --- | --- | --- |
| ANOVA 1 | | | | | | |
| Asian identity | 1 | 0.20 | 0.20 | 0.22 | 0.638 |  |
| Condition | 2 | 4.48 | 2.24 | 2.49 | 0.084 |  |
| Condition x Asian | 2 | 0.42 | 0.21 | 0.23 | 0.792 |  |
| Residuals | 468 | 421.48 | 0.90 |  |  |  |
| ANOVA 2 | | | | | | |
| Latinx identity | 1 | 0.01 | 0.01 | 0.01 | 0.903 |  |
| Condition | 2 | 4.48 | 2.24 | 2.49 | 0.084 |  |
| Condition x Latinx | 2 | 0.55 | 0.27 | 0.30 | 0.739 |  |
| Residuals | 468 | 421.54 | 0.90 |  |  |  |
| ANOVA 3 | | | | | | |
| First Gen. Status | 1 | 0.85 | 0.85 | 0.96 | 0.328 |  |
| Condition | 2 | 5.14 | 2.57 | 2.89 | 0.057 |  |
| Condition x First Gen. Status | 2 | 0.40 | 0.20 | 0.22 | 0.799 |  |
| Residuals | 465 | 413.44 | 0.89 |  |  |  |

*Note*. SS = sum of squares. MS = mean square.

**Table S6**

*Average Effort (Standardized) by Condition and Demographic Group*

| Demographic Group | Behavioral  Self-Kindness | | Cognitive  Self-Kindness | | Control |  |
| --- | --- | --- | --- | --- | --- | --- |
|  | M(SD) | | | | |  |
| Asian | -0.06 (0.92) | 0.31 (0.94) | | -0.51 (1.00) | | |
| Non-Asian | -0.01 (0.96) | 0.56 (0.81) | | -0.23 (0.95) | | |
| Latinx | 0.07 (0.86) | 0.72 (0.78) | | -0.18 (0.97) | | |
| Non-Latinx | -0.07 (0.96) | 0.33 (0.89) | | -0.45 (0.98) | | |
| First-Generation | 0.00 (0.87) | 0.65 (0.90) | | -0.3 (1.00) | | |
| Continuing-Generation | -0.06 (0.99) | 0.29 (0.84) | | -0.45 (0.97) | | |

**Table S7**

*Two-Way ANOVA of Effects of Condition and Demographic Group on Effort (Standardized)*

|  | *df* | SS | MS | *F* | *p* |
| --- | --- | --- | --- | --- | --- |
| ANOVA 1 | | | | | |
| Asian identity | 1 | 4.25 | 4.25 | 4.91 | **0.027** |
| Condition | 2 | 50.52 | 25.26 | 29.23 | **< .001** |
| Condition x Asian | 2 | 1.34 | 0.67 | 0.78 | 0.461 |
| Residuals | 468 | 404.49 | 0.86 |  |  |
| ANOVA 2 | | | | | |
| Latinx identity | 1 | 7.06 | 7.06 | 8.21 | **0.004** |
| Condition | 2 | 50.52 | 25.26 | 29.41 | **< .001** |
| Condition x Latinx | 2 | 1.02 | 0.51 | 0.59 | 0.553 |
| Residuals | 468 | 402.00 | 0.86 | NA | NA |
| ANOVA 3 | | | | | |
| First Gen. Status | 1 | 4.06 | 4.06 | 4.69 | **0.031** |
| Condition | 2 | 50.75 | 25.37 | 29.28 | **< .001** |
| Cond. x First Gen. | 2 | 1.99 | 0.99 | 1.15 | 0.319 |
| Residuals | 465 | 402.96 | 0.87 | NA | NA |

*Note*. SS = sum of squares. MS = mean square. Cond. = condition. First gen. = first generation status. Bolded text indicates statistically significant predictors (*p* < .05).

**Table S8**

*Estimated Marginal Means and Standard Errors in Effort (Standardized) by Demographic Group*

| Demographic Group | MM (SE) |
| --- | --- |
| Asian | -0.09 (0.06) |
| Non-Asian | 0.11 (0.06) |
| Latinx | 0.20 (0.08) |
| Non-Latinx | -0.07 (0.05) |
| First-Generation | 0.12 (0.06) |
| Continuing-Generation | -0.07 (0.06) |

Note. MM = Estimated marginal means.

SE = Standard error.
